# Polymeric Hydrogels for Intervertebral Disc Replacement/Integration: Playing with the Chemical Composition for Tuning Shear Behavior and Hydrophilicity

**DOI:** 10.3390/gels9110912

**Published:** 2023-11-17

**Authors:** Devid Maniglio, Elia Bissoli, Emanuela Callone, Sandra Dirè, Antonella Motta

**Affiliations:** 1 BIOtech Research Center, Department of Industrial Engineering, University of Trento, Via Delle Regole 101, 38123 Trento, Italy; devid.maniglio@unitn.it (D.M.); antonella.motta@unitn.it (A.M.); 2 “Klaus Müller” Magnetic Resonance Lab., Department of Industrial Engineering, University of Trento, Via Sommarive 9, 38123 Trento, Italy

**Keywords:** PEG-based hydrogels, solid-state NMR, chain molecular dynamics, shear properties, IVD remediation

## Abstract

Damages to the intervertebral disc (IVD) due to improper loading or degeneration result in back pain, which is a common disease affecting an increasing number of patients. Different strategies for IVD remediation have been developed, from surgical treatment to disc replacement, by using both metallic and non-metallic materials. Hydrogels are very attractive materials due to their ability to simulate the properties of many soft tissues; moreover, their chemical composition can be varied in order to assure performances similar to the natural disc. In particular, for the replacement of the IVD outer ring, namely, the *anulus fibrosus*, the shear properties are of paramount importance. In this work, we produced hydrogels through the photo-induced crosslinking of different mixtures composed of two hydrophilic monofunctional and difunctional polymers, namely, poly(ethyleneglycol) methyl ether methacrylate (PEGMEMA) and poly(ethyleneglycol) dimethacrylate (PEGDMA), together with a hydrophobic molecule, i.e., tert-butyl acrylate (tBA). By changing the ratio among the precursors, we demonstrated the tunability of both the shear properties and hydrophilicity. The structural properties of hydrogels were studied by solid-state nuclear magnetic resonance (NMR). These experiments provided insights on both the structure and molecular dynamics of polymeric networks and, together with information obtained by differential scanning calorimetry (DSC), allowed for correlating the physical properties of the hydrogels with their chemical composition.

## 1. Introduction

The intervertebral disc (IVD) is a largely avascular and aneural fibro-cartilaginous structure in the human body and, together with two neighboring vertebrae, forms the functional motion unit of the human spine. The disc can be divided into three regions: the central ones called the *nucleus pulposus* (NP), the outer ring called the *anulus fibrosus* (AF) and the cartilaginous endplates, which anchor the discs to the adjacent vertebrae [1]. On average, the IVD sizes are 40 mm in diameter and 7 to 10 mm in thickness, depending on age and gender [2].

The intervertebral disc has a multi-component structure: from the outer fibrous multi-layered composite (AF) to an inner compact and continuous hydrogel (NP), with a transition region having intermediate properties and contributing to dispersing the forces from the NP toward the endplates as well as the AF. The IVD components have different compositions, but the main constituents can be identified as proteoglycans and collagen fibrils (mainly types I and II).

The NP is made of a hydrogel based primarily on type II collagen and proteoglycans, with a water content of 66% to 86%. The proteoglycans include aggrecan and versican, which are bound to hyaluronic acid. Aggrecan is mainly responsible for the water content within the NP. The integrity of the NP is maintained by the cells responsible for the synthesis of the macromolecules constituting the low-density cells able to synthesize the extracellular matrix (ECM) molecules [3].

The AF is instead composed of concentric layers of differently oriented collagen fibers to enable multidirectional resistance and contrast the lateral expansion of the NP when subjected to a vertical load. The AF varies its main composition from the outside (mainly collagen type I) to the inside region (collagen type II and proteoglycans) [4] and presents 60 to 85% water content [5,6].

The shear properties of the AF are important for controlling and limiting motion between vertebrae during bending and twisting of the spine. The magnitude of the complex shear modulus (|G*|) is the shear modulus under dynamic conditions, and the phase shift angle between stress and strain (δ) is a relative measure of viscoelasticity. The complex shear modulus of the AF has a range of 0.10–0.28 MPa and δ has a range of 9–35 deg depending on the frequency and shear strain amplitude [7,8,9,10].

Damages due to improper loading or degeneration of the intervertebral disc result in back pain, a common pathology that affects an increasing number of patients, due to an increase in average age [11,12]. Tears are usually the result of sudden, massive stresses when the body is in an inappropriate posture leading to an overload of the disc. Acute tearing or chronic degeneration of the posterior lamellae of the *anulus fibrosus* allows for deformation and herniation of the *nucleus pulposus*. The disc usually prolapses just lateral to the posterior longitudinal ligament and can compress one or two spinal nerves unilaterally. Much less commonly, the prolapse is central, in the midline posteriorly. If the damaged *anulus* ruptures completely, some of the nuclear tissue may escape into the vertebral and the root canals. This sequestered material may migrate within the canals and cause nerve compression at spinal levels distant from those of the disc rupture. The disc material itself may have an irritative effect on the spinal nerve. These conditions may put pressure on the spinal cord and nerves, leading to pain and possibly affecting nerve function [13].

On the basis of the seriousness of IVD degeneration or damages, several clinical strategies have been developed, even if surgical treatment does not address the underlying problems, such as the biological and structural deterioration of the disc [14]. Some techniques imply the physical confinement of the IVD, addressed as dynamic stabilization (such as Graf and Dynesys) [15], or the induced retraction of the annular collagen stimulated by intradiscal electrothermal therapy (IDET) [16]. Some others imply a more invasive approach, like spinal fusion or total or partial disc replacement [17].

A total disc replacement can be performed using metallic or non-metallic materials, or a combination of the two. While metallic disc replacements take advantage of their high strength and the capability of withstanding loads and fatigue cycles, they limit body movement. Moreover, the continuous friction between the endplates and the vertebrae can cause the accumulation of debris, triggering tissue inflammation [18].

Non-metallic devices usually rely on finding polymers or elastomers with mechanical performances similar to the natural disc. Due to their ability to simulate the nature of many soft tissues, hydrogels are highly attractive materials for developing synthetic ECM analogs [19,20,21]. Furthermore, many hydrogels can be formed under mild, cytocompatible conditions and can be modified to introduce cell adhesion ligands or match the desired viscoelasticity or degradability [22,23]. They can also be functionalized with growth factors to stimulate the growth of cells and support tissue repair and regeneration. Another key feature of these systems is their capability to be used as bioinks for 3D bioprinting techniques in order to create complex shapes or hybrid systems in combination with other polymers [24,25] and their possibility of being designed even at the nanoscale [26]. In this respect, hydrogels are so versatile because by varying the chemical composition and material types, the molecular weight and crosslinking degree and the solid contents and functionalization, they can be used to mimic the mechanical behavior of the IVD [27,28,29].

An important class of hydrogels suitable for tissue-engineering applications and with possible applications in IVD structures replacement/integration are polyethylene glycol (PEG) derivatives: synthetic, highly hydrophilic and biocompatible polymers characterized by their negligible recognition by the immune system, a property that gives them stealth-like behavior [30,31,32,33]. PEG-based hydrogels possess highly tunable mechanical properties (shear, tensile and compression, and tensile) as well as water-swelling behavior, resembling the properties of several human tissues, in particular those with low cellular content, such as cartilage. PEG-based hydrogels have been proposed in combination with other synthetic or natural polymers or moieties, like polylactic acid or hyaluronic acid, tripeptide Arg-Gly-Asp (RGD) sequences or enzyme-sensitive peptides, to tune and boost cell adhesion and proliferation [34,35,36,37,38,39]. By the way, the overall mechano-rheological and fluid exchange properties can be tailored in principle, just by the selection of the molecular weight and hydrogel parameters, tuning the behavior of the embedded cell clusters in terms of morphology, metabolite exchange or extracellular glycosaminoglycan synthesis [40].

Moreover, despite the fact that unreacted species, oligomers and initiators might contaminate and leach out of the hydrogel during usage leading to toxicity issues [41], the possibility of synthesizing a PEG-based polymer from polymer subunit combinations as well as the use of a cytocompatible photoinitiator, such as LAP (Lithium phenyl-2,4,6-trimethylbenzoylphosphinate), can represent an escape route for adverse effects mitigation and safe translation to the clinic.

The presented work focuses on the research on chemical hydrogels [42] resulting from the combination and photo-induced reaction of two hydrophilic polymers, poly(ethyleneglycol) methyl ether methacrylate (PEGMEMA) and poly(ethyleneglycol) dimethacrylate (PEGDMA) together with a hydrophobic molecule (tert-butyl acrylate (tBA)). The combination of a bifunctional PEG, with either a hydrophilic or/and a hydrophobic molecule, allows for large freedom in the selection of combinations, enabling not only the tuning of the hydrophilic behavior but also having a direct impact on the expected thermo-mechanical properties. Different compositions have been characterized by assessing their rheological behavior together with their capacity to retain water. These characteristics have then been compared with the results coming from solid-state NMR analyses that, together with scanning calorimetry, contribute to the definition of the structural description of the hydrogels, with the unprecedented possibility of properties tuning, allowing the design of a universal, easy bio-functionalizable platform possessing tunable molecular weight, mechanical properties, hydrophilicity and crosslinking degree for addressing *anulus fibrosus* repair or regeneration strategies.

## 2. Results and Discussion

The polymeric hydrogels composed of tBA, PEGDMA and PEGMEMA were prepared by photopolymerization. The as-prepared samples with the nominal compositions summarized in Table 1 appeared as homogeneous, flexible and nearly transparent discs (Figure S1). The results of the different physical–chemical analyses on the eight samples of the three-component hydrogel materials are here detailed.

### 2.1. Water Uptake/Swelling Behavior

**Figure 1:**
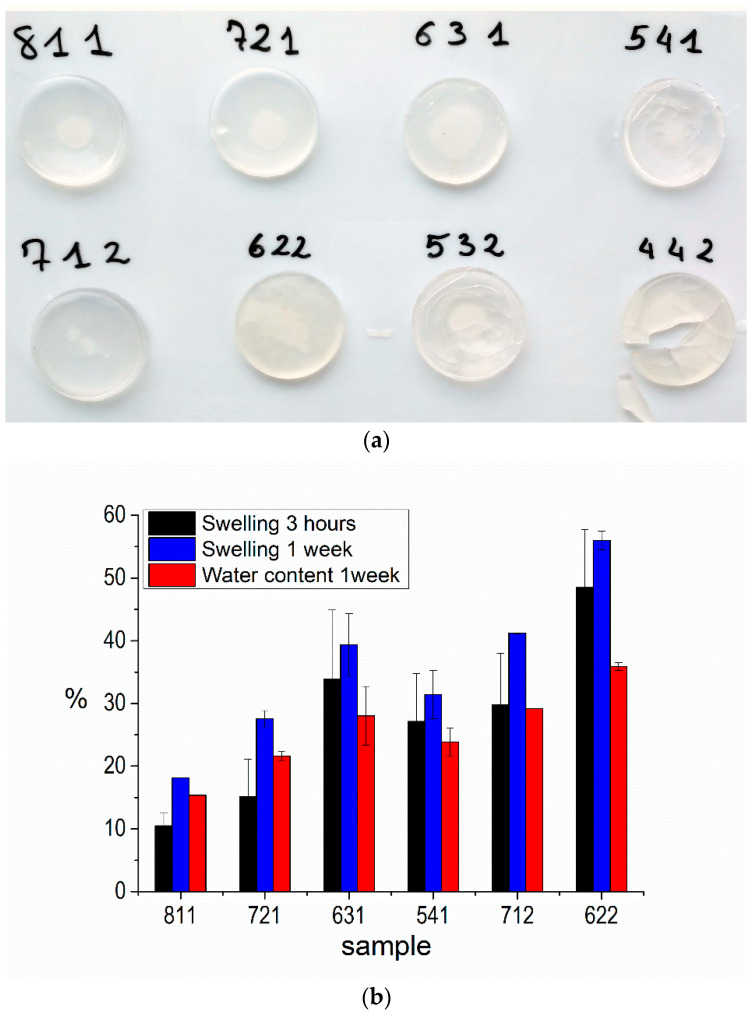
(**a**) Images of the samples after 1 week of immersion in water and (**b**) swelling (after 3 h and 1 week) and water content (after 1 week) of selected compositions. The three-digit labels refer to the nominal ratio tBA:PEGDMA:PEGMEMA, as detailed in Table 1.

PEG-based materials possess the ability to absorb and retain large amounts of water, which consequently should be considered as an essential component. For this reason, all the specimens were first tested to evaluate the water uptake and swelling behavior in order to assess the range of nominal compositions of the materials approaching the desired performances [17,42]. Figure 1 shows the appearance of the samples after 1 week of swelling, and the water content and swelling ratio, which were calculated by Equations (2) and (3) respectively (Section 4.3). From Figure 1a, it is clear that some compositions should be discarded due to unavoidable fragility, such as those with lower tBA content (532 and 442), which resulted in cracking after a few days of soaking. Due to the mass loss and inability to maintain the shape, some analytical results on these samples are not discussed further on.

The swelling behavior at different intervals, together with the water uptake, is reported in Figure 1b and, as expected, is clearly affected by the amount of both PEGMEMA and PEGDMA. Indeed, it is noticeable that increasing the amount of the PEG-based compounds in the hydrogels determines the swelling from 10 to almost 50% after 3 h and from 15 to 55% after one week of immersion in water. At the same time, the overall water content ranges from 16 to 36%. Furthermore, samples with higher PEGMEMA content display a higher ability to interact with water, as can be seen by comparing sample 712 with sample 721. The trend is not respected by sample 541, but probably the measurements are affected by the appearance of some cracks (Figure 1a), as previously observed for samples 532 and 442.

### 2.2. DSC Measurements

Differential scanning calorimetry analyses were performed on the pure reagents (tBA, PEGDMA and PEGMEMA) and the six samples selected from the swelling analysis. The DSC main data for tBA, PEGMEMA and PEGDMA are summarized in Table S1. The tBA has a large evaporation peak with the onset at about 24 °C and the minimum at about 92 °C, while PEGMEMA shows a melting peak centered at about 36 °C. PEGDMA shows an evaporation peak centered at about 70 °C, attributable to the evaporation of the adsorbed water, due to its absence in the second scan, and a melting peak at 3 °C (6 °C in the second run), which is out of the temperature range of interest for this work.

**Figure 2:**
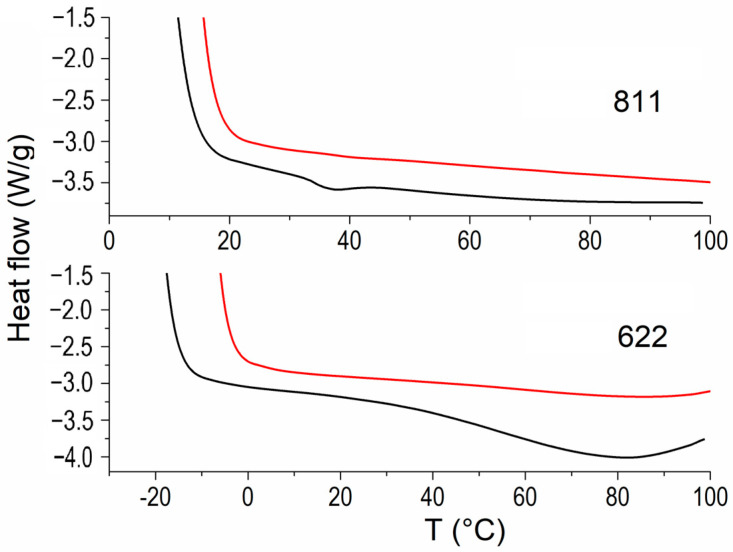
Double-run DSC analyses on 622 and 811 samples (black: 1st run; red: 2nd run).

Figure 2 reports the DSC diagrams (first and second heating scans) of samples 811 and 622 that are representative of the 10 and 20%wt PEGMEMA- and PEGDMA-containing samples.

The as-prepared samples appear stable in the tested temperature range, with no significant weight variation detected, and their thermal behavior speaks of nearly complete polymerization. Sample 811 is characterized by a single small peak centered at about 37 °C, which is compatible with a melting signal of the PEGMEMA, which does not recrystallize in the second run. Oppositely, sample 622 shows in the first scan a broad band centered at about 77 °C that shifts to about 88 °C and reduces in intensity in the second scan, which can be attributed to the overlapping of the water evaporation band and the PEGMEMA melting band.

The DSC diagrams of the other samples (namely, 721, 631, 541 and 712), reported in Figure S2, show that increasing the PEGDMA content generally leads to the increase in the intensity of the band associated with water evaporation, and this effect is even more pronounced with an increasing PEGMEMA amount.

### 2.3. Dynamic Shear Measurements

**Figure 3:**
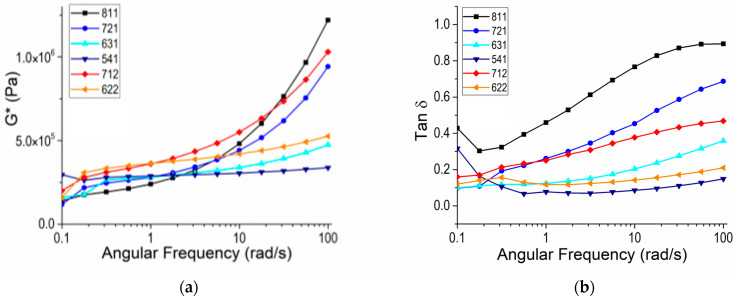
(**a**) Complex modulus and (**b**) tan δ as a function of the frequency measured on the samples after 3 h soaking in water at 37 °C.

Rheological analyses were performed to evaluate the effect of the different components on the elastoplastic response of the final crosslinked polymer and better matching the mechanical behavior of the *anulus fibrosus*. The complex modulus (G*) and dumping (tan δ) were measured through the dynamic shear analyses performed on the six samples coming from the previous section. In Figure 3, we report the results measured on the samples after 3 h soaking in DI water, which are well representative of the general behavior of the materials, even for prolonged immersion.

All the samples revealed a viscoelastic behavior, with the G* values ranging from 1 × 10^5^ Pa to 1.3 × 10^6^ Pa. At low frequencies (<1 rad/s), all the samples appear similar in terms of complex shear stiffness, with mostly elastic behavior (tan δ < 0.4). The differences are more evident at medium and high frequencies (>1 rad/s), where the samples with higher tBA content (811, 712 and 712) show a strong increase in the G* values, and similarly of tan δ, with respect to the samples with the tBA equal or less than 60%wt, for which the parameter values remain quite low. Moreover, at 100 rad/s, throughout the 10%wt PEGMEMA series (811, 721, 631 and 541), both the G* and tan δ values tend to decrease with the increase in the PEGDMA amount. A similar behavior is observed comparing the samples with 20%wt PEGMEMA, namely, 712 and 622. Finally, sample 541 with the highest PEGDMA amount shows values of tan δ almost constant over all the tested frequencies, together with an increase in G* only at very high frequencies, which corresponds to an increase in the conservative modulus, reflecting in an almost exclusive elastic behavior.

Allowing the networks to reach their swelling equilibrium, after 1 week of soaking, results in a general slight increase in G* (data not shown). G* increases more for the samples with higher tBA content, such as 811 or 712. The tan δ values follow the same trend, with higher values for the samples with higher tBA content, even if to a lower extent when compared to the corresponding values measured after 3 h of soaking.

### 2.4. Solid-State Nuclear Magnetic Resonance Study

The structural differences induced at the molecular level by the reagent ratios were investigated in depth by ^13^C CPMAS NMR experiments [43,44,45]. Therefore, the spectra were recorded on all the produced hydrogels, despite their swelling and rheological behavior.

**Figure 4:**
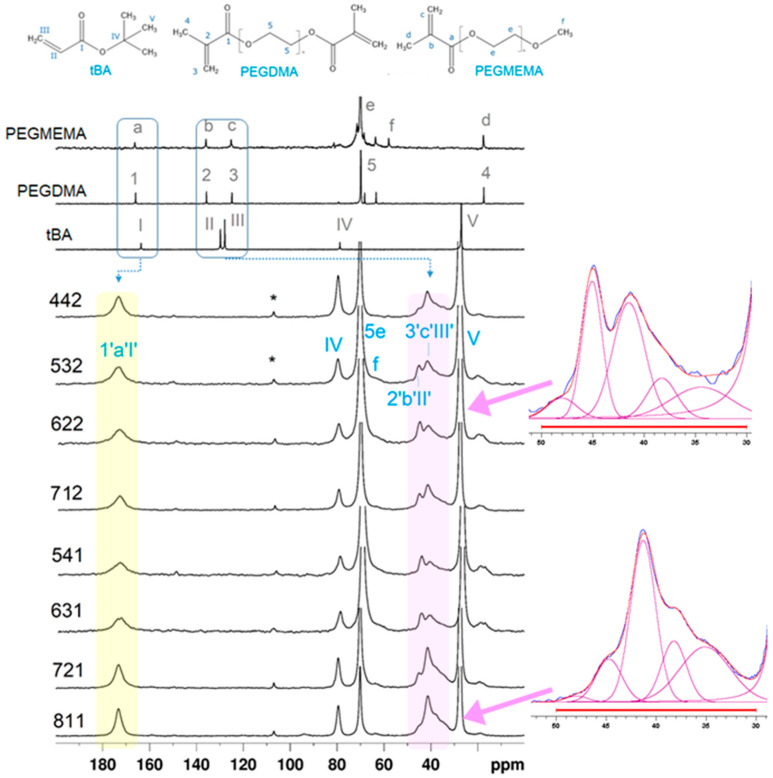
(**top**) Carbon labeling of the precursors. (**bottom**) ^13^C CPMAS NMR spectra of the samples. Peaks marked with * are spinning sidebands. The most intense peaks due to methylene in the long chains are cut in order to give visibility to more interesting features. On the right, two examples of the profile-fitting analysis of the 40 ppm resonance are shown.

The hydrogel spectra are shown in Figure 4, together with those of the pristine reagents, which are presented for the sake of clarity in the upper part of the figure. The peaks are labeled according to the schemes in Figure 4 (top) [46,47].

**Scheme 1:**
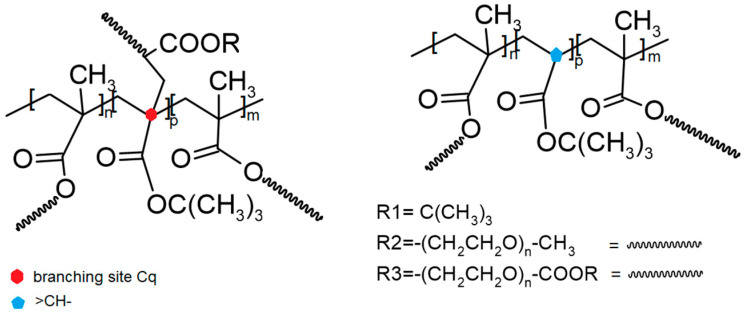
For the sake of clarity, the scheme presents a simple picture of the main chain formed by photopolymerization, but it can not be considered a strict representation of the hydrogels structure; on the left, the formation of branching sites from carbons II of tBA is highlighted; the possible results of the polymerization reactions of tBA with PEGMEMA and PEGDMA precursors are shown on the right.

Figure 4 shows that in all the samples, the polymerization appears complete, in agreement with (i) the absence of the resonances due to the C=C double bonds in the region 120–140 ppm; (ii) the downfield shift of the C=O resonance with respect to the carbonyl chemical shift detected in the spectra of pure reagents and (iii) the presence of a broad band in the region 30–50 ppm, which results from the signal overlapping of all the new bridging aliphatic carbons produced by polymerization (Scheme 1, right).

To assess the stability of the samples over time, the ^13^C CPMAS NMR spectra were also recorded after five months on the specimens stored in the laboratory without special precautions. The carbon spectra are superimposable to those of fresh samples (Figure S3), meaning that there is no detectable effect of aging on the molecular structure.

It is worth noting that the signals due to both the C=O (around 173 ppm) and the new aliphatic signals (around 40 ppm) change in shape with the changing of the reagents’ ratio (Figure 4). Therefore, these two regions were analyzed more in detail.

**Figure 5:**
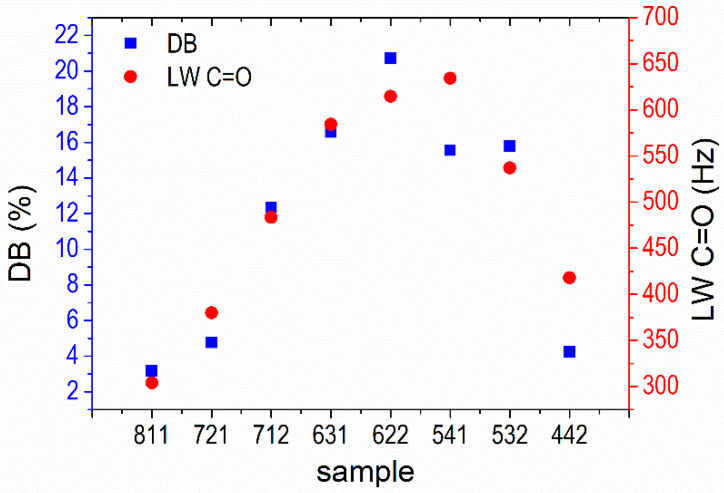
Linewidth (LW) of the C=O resonance and DB% (calculated from Equation (S1) in Supplementary Materials) as a function of samples composition.

At first, it should be noticed that the carbonyl resonance at 173 ppm (**I**/**1**/**a**) modifies its linewidth among the samples (Figure 4). Because this resonance is composed of more than 80% of the tBA carbonyl groups and all the reagents contribute with one or two units per molecule, it could be used as a sensitive probe of the structural variability. Its Lorentzian lineshape could be satisfactorily fitted with one component that changes the overall linewidth (LW) as a function of the sample composition (Table S2 and Figure 5).

Secondly, according to the molecular labeling in Figure 4, the double-bond reaction of the three reagents gives rise to methylenes (**III′,3,c′**), methines (**II′**) and quaternary carbons (**2′,b′**). These functional groups form the skeleton of the hydrogel chains. Additionally, the photopolymerization also induces branching, when the reaction involves site **II** of tBA, forming another quaternary carbon site (**II″**) [48,49]. A simple picture of the functional groups involved in the polymerization is presented in Scheme 1.

All the produced functional groups give rise to the resonance around 40 ppm, resulting in an overlapped band, whose fitting is proposed in the insets of Figure 4. A profile-fitting analysis is useful not only for the identification of the resonances but also for calculating the degree of branching (DB%) of the materials, which indicates the presence of both side-chain and crosslinking points and highlights the polymeric chain organization. According to the literature [48,50], the DB% and the associated error can be calculated by Equations (S1) and (S2) from the area of the new signals belonging to polymerized tBA, as explained in detail in the Supplementary Materials (Figure S4 and Table S2).

Figure 5 reports the calculated values of both the LW_C=O_ and DB% parameters for all the samples. Interestingly, they follow the same trend among the samples: the values increase when reducing the tBA content, with a maximum at 60%wt tBA, and then they decrease. 

Finally, it is well known that the chemical composition also affects the dynamic of the polymeric network at the molecular level, which in turn influences the macroscopic mechanical properties of the materials [51,52,53]. The local chain dynamics can be assessed by additional ^13^C NMR analyses, evaluating the trend of the signal intensity (i.e., magnetization) as a function of the contact time (Figure S5) [43,44]. The magnetization curves are usually characterized by two components, exponential growth followed by exponential decay. The trends are determined by the kinetics and dynamics of the protons-to-carbons cross-polarization process. The simplest fitting model is represented by Equation (3) reported in Section 4.3 [44]. The initial growth at short contact times is governed by the ^1^H-^13^C polarization transfer (T_CH_), which depends on the mutual C–H distance and H atoms’ density in the proximity of a given C atom (ms range) and is disturbed by possible functional group mobility. Instead, the subsequent decay depends on the spin–lattice relaxation in the rotating frame (*T*_1*ρ*(*H*)_) that is influenced by the local lattice features. 

**Figure 6:**
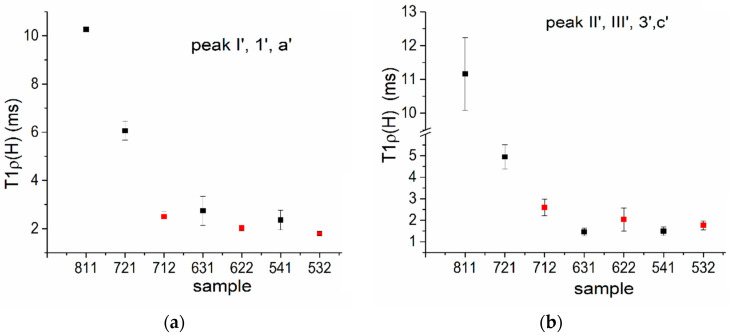
Trends of the extrapolated *T*_1*ρ*(*H*)_ of the ^13^C VCT NMR magnetization curves for the main resonances C=O, i.e., peak I′,1′,a′ (**a**), and CH-CH_2_, i.e., peak II′,III′,3′,c′ (**b**), of all the samples. Black spots refer to the series with 10%wt of PEGMEMA, whereas the red spots refer to the series with 20%wt of PEGMEMA.

It is worth mentioning that for all the samples, the curves (Figure S5) are well fitted with both single exponential growth and decay. Focusing on the decay part of the magnetization curves, i.e., on the values of the spin–lattice relaxation time *T*_1*ρ*(*H*)_, the most sensitive functional groups in our samples are the previously discussed C=O (**I′**/**1′/a**) and the formed -CH_2_-CH- groups in the polymeric chain (**II′**/**III′/3′/c′**). The *T*_1*ρ*(*H*)_ values of the two functional groups are reported in Figure 6a,b and highlight the different behavior of samples 811 and 721 with respect to all the others. As a matter of fact, reducing the tBA has a strong impact on the local molecular dynamics in the studied kHz range for the representative signals. For both signals, the exponential decay branch decreases faster and faster, especially going from 80%wt to 60%wt of tBA.

In addition, the different behavior shown by the 721 and 712 samples, characterized by the same percentage of tBA but a different amount of PEGMEMA, should be noted. 

### 2.5. Discussion

In this work, polymeric hydrogels with variable composition were studied in order to propose a selection of materials with tunable crosslinking, hydrophilicity and shear mechanical properties, suitable for the intervertebral disc remediation/integration, in particular for mimicking the performances of the *anulus fibrosus* (AF). Accordingly, eight types of samples of three-component polymeric hydrogels based on photosensitive precursors, namely, tBA, PEGDMA and PEGMEMA, were prepared by photopolymerization. The reagents’ ratio was varied from a maximum of 80 to a minimum of 50%wt of tBA, and two series of samples were produced, changing the amount of PEGMEMA from 10 to 20%wt, to assess the influence of the chemical composition on the properties of the final materials.

Solid-state NMR experiments were performed to characterize in detail the molecular structure of the materials, highlighting in particular the changes induced by the reagents’ ratio. The NMR study proved that the hydrogels are homogeneous materials with fully polymerized components, according to the ^13^C CPMAS spectra (Figure 4) and in agreement with the DSC results (Figure 2 and Figure S2).

The variations in both the C=O lineshape and the branching degree (Table S2 and Figure 5) suggest that chain packing and crosslinking are dependent on the hydrogels’ composition. According to Figure 5, both structural parameters follow the same trend among the samples, so they can be indifferently used to draw correlations with the macroscopic features of the hydrogels. As a matter of fact, in the polymer field, the carbonyl peak is frequently used as a probe of chain conformation [54], because its lineshape variation could be related to the different tacticity, chain packing and molecular rigidity induced by the polymerization process in relation to the sample composition. This is a very interesting feature, because polymer stereochemistry is strictly correlated to the physical–chemical properties, for example, the glass transition [47,55]. In addition, the branching points interfere with an ordered alignment of the chains and are necessary for the creation of crosslinks, thus affecting the tridimensionality of the polymer network and the physical properties, such as the melting temperature [56,57].

It is clear from Figure 5 that the carbonyl linewidth (LW) increases as the amount of tBA decreases; the highest LW values are found for samples with 50–60%wt of tBA. This result can be explained by the high structural disorder that in samples such as 622 and 631 is caused by the higher degree of branching. More generally, as might be expected, the highest DB values are obtained with high amounts of both PEGDMA and PEGMEMA, and preferably for the 20%wt PEGMEMA series compared with the 10%wt one. Furthermore, it is worth noting that an even smaller amount of tBA leads to the decrease in both the LW and DB (sample 442).

The resonances due to the C=O (173 ppm) and the CH_2_ groups created by the polymerization (40 ppm) also showed interesting trends in the study of the molecular dynamics, which may add further information on the relation between functional properties and chemical composition. These are resonances of particular interest, because it is well recognized that the carbonyl group reflects the overall chain behavior [52,58] while the methylene response provides insight into the organization of the polymer network.

As described in the previous section, we focused in particular on the relaxation dynamics in the kHz range through the evaluation of the decay constant T_1ρ(H)_ of the protons, because it is a volume property averaged over a distance of ca. 2 nm and provides information on the relative mobility of the H atoms. In homopolymers, high T_1ρ(H)_ values are related to long chains or rigid/crystalline materials and low values are generally associated with amorphous/mobile phases or even heterogeneous domains [58].

For all the samples, the decay of the ^13^C VCT magnetization curves (Figure S5) can be fitted with a single exponential, confirming the homogeneity of the produced materials.

Figure 6 shows the trends of the extrapolated T_1ρ(H)_ of the most sensitive functional groups, namely, C=O (**I′**/**1′/a**) and the formed -CH_2_-CH- groups in the polymeric chain (**II′**/**III′/3′/c′**), as a function of the samples’ composition. For the studied resonances, the T_1ρ(H)_ parameter reduces with the reduction in tBA, suggesting higher disorder/mobility of the functional groups due to the copolymerization: as a matter of fact, C=O is close to and -CH_2_- is located at the polymerization site. Reducing the amount of tBA increases the probability of all the possible copolymerization combinations and the creation of branching, as already noticed by studying both the DB% and LW, and this should lead to more disordered chain packing.

The samples 811 and 721 show very different T_1ρ(H)_ values compared with the other samples, and this result can be explained with a more rigid network, both at the molecular and macroscopic levels. In addition, these two samples are characterized by a reduced branching degree and less disordered chain packing, according to the results shown in Figure 5.

Finally, the comparison between samples 721 and 712 may also unravel the role of PEGMEMA, which is a monomer characterized by a longer PEG backbone than PEGDMA and acts as a plasticizer. It is worth mentioning that all the NMR data highlight the very different behavior showed by samples 721 and 712, despite the same amount of tBA. In detail, sample 712 presents a significantly higher C=O LW, DB and disorder/chain mobility, according to a lower T_1ρ(H)_ value, with respect to the same functional groups in sample 721.

The different structural properties observed in hydrogels with varying contents of tBA, PEGDMA and PEGMEMA are reflected in the different hydrophilicity and shear mechanical properties. As detailed in the previous section, apart from samples 442 and 532 that should be discarded due to cracking upon swelling (Figure 1a), the samples with higher content of PEGDMA and PEGMEMA display a higher swelling degree and water uptake (ranging from 16 to 36%), according to the hydrophilic character conferred by the PEG-based compounds as compared to tBA (Figure 1b), but still far from the natural values for the AF. The higher capability of the samples with a higher PEG-based component to incorporate water is also proved by the comparison of the DSC curves of the 622 and 811 samples (Figure 2). Furthermore, for the same tBA content, PEGMEMA appears to be more efficient in interacting with water, as can be seen by comparing samples 712 and 721 (Figure 1b), due to both the longer PEG backbone and the presence of only one acrylate group, which results in a lower degree of crosslinking in the hydrogel network after polymerization.

The reagents’ ratio strongly affects the shear mechanical behavior of the hydrogels (Figure 3). This is especially clear from the trends of G* and tan δ, which both experience a strong increase at medium and high frequencies when the tBA amount is above 70%wt, thus reflecting a higher energy dissipation potential. Oppositely, among all the samples, samples 631, 622 and 541 are characterized by a more elastic behavior, in consequence of the reduced amount of tBA and the improved hydrophilicity of the networks. As observed previously, the PEGDMA/PEGMEMA ratio plays a significant role, as the reduction in PEGDMA content leads to an increase in the damping capacity of the system, owing to the increased chain mobility, in agreement with the molecular dynamics study. The results reported by Iatridis et al. [59] on the dynamic shear properties of specimens taken from non-degenerated human discs can be used for a preliminary assessment of the performance of the hydrogels produced in this work. The human AF samples display the increase in G* and tan δ with increasing frequencies, in agreement with the behavior of PEG-based hydrogels [59], but the dependence on the frequency of stress appears to be smoother. At frequencies equal to 0,1 Hz, 1 Hz (typical of breathing or vertebral adjustment during standing or sitting stances) and 10 Hz (representative of external vibrations, such as that of industrial machines), the human AF displays tan δ values of about 0.3–0.35 and G* values of 1–1.5 × 10^5^ Pa [59], which are in quite good agreement with the values obtained for the samples with low tBA content, such as 631, 622 and 541.

**Figure 7:**
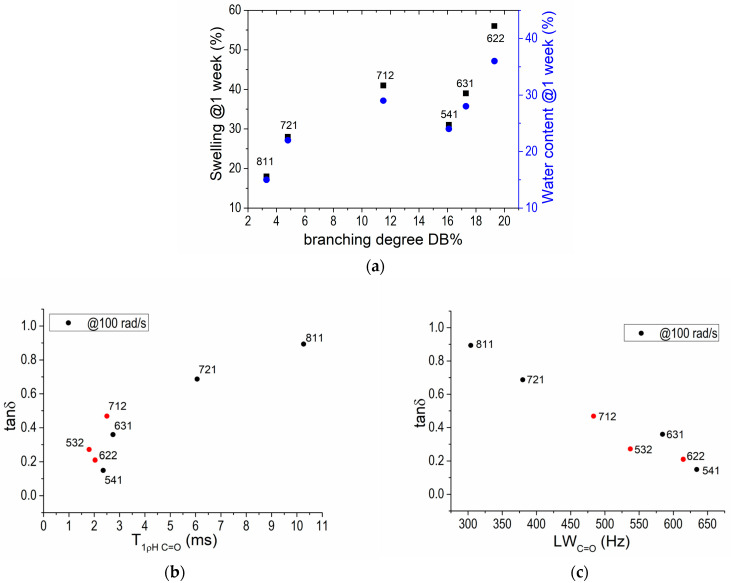
Trends of both swelling ability and water uptake with respect to the DB% (**a**), tan δ at 100 rad/s vs. the carbonyl LW (**b**) and vs. the spin–lattice relaxation time (**c**). In (**b**,**c**), black spots refer to the series with 10%wt of PEGMEMA, whereas the red spots refer to the series with 20%wt of PEGMEMA.

The structural influence on the measured properties is well pointed out in Figure 7, which presents the correlations among the results obtained from the detailed NMR study and the discussed swelling, water uptake and shear behaviors. In detail, Figure 7a shows the trends of swelling and water content vs. the branching degree, while the tan δ values are reported against the LW of the C=O resonance in Figure 7b. Accordingly, networks presenting a high branching degree and structural disorder are characterized by a high swelling ability, water uptake and damping capacity. The performances of the hydrogels also well correlate with the chain mobility in the networks, according to Figure 7c that presents the trend of tan δ vs. T_1ρ(H)_.

By combining all the results, it appears that sample 622 shows the best performances in terms of swelling, water uptake capability and elasticity due to the high branching degree and chain mobility, favored by the high amount of the long PEG-chain, and high structural disorder. A high content of tBA, such as for samples 811 and 721, produces samples that are stiff, rigid and hydrophobic. Therefore, good elasticity and hydrophilicity can be obtained with 40–50%wt of mono- and bifunctional PEG-based polymers because these are able to create crosslinked hydrophilic networks with high molecular mobility, good swelling and damping behaviors.

## 3. Conclusions

Hydrogel samples with different compositions were obtained by the photo-crosslinking of the mixtures made of mono- (PEGMEMA) and difunctional (PEGDMA) PEG-based polymers and tBA. The DSC results point out the different water uptake and swelling capabilities of the samples, directly correlating the tBA content with the hydrophobicity. The dynamic shear measurements pointed out that, at medium and high frequencies, the tBA-rich samples (811, 712 and 712) are characterized by large values of both G* and tan δ, reflecting higher stiffness and energy dissipation potential than samples with a larger content of PEG-based polymers. Moreover, the relative ratio between the PEGDMA and PEGMEMA affects the shear mechanical properties of the hydrogels.

The extensive solid-state NMR characterization of the hydrogels through the calculation of the branching degree and linewidth of the C=O resonance allowed for elucidating the polymeric chain organization and provided information on the chain dynamics at the molecular level. The correlation of the NMR parameters with the physical properties highlighted that the best performances in terms of the swelling, water uptake capability and elasticity are associated with both a high branching degree and chain mobility. These features are favored by both a high amount of PEG-based polymers and high structural disorder.

In conclusion, the present study suggests a methodology to evaluate the trends’ property/composition to tailor the photopolymerized hydrogels according to some of the key properties required for annulus fibrosus remediation, constituting a robust base for solutions development. In particular, with the aim of designing a prosthetic device, there is space for extending the research in the direction of properly modeling the radial loads deriving from the *nucleus polposus* compression. The proposed approach can hasten the development of composite materials that bolster the radial mechanical strength while maintaining other essential characteristics.

In this context, grasping the link between the material’s structure and its physical attributes is essential for swiftly pinpointing the optimal formulation and processing techniques tailored to the end use.

## 4. Materials and Methods

### 4.1. Materials

Tert-butyl acrylate (tBA, MW 128.17 g mol^−1^), poly(ethyleneglycol) methyl ether methacrylate (PEGMEMA, MW 950 g mol^−1^, Tm = 33–38 °C), poly(ethyleneglycol) dimethacrylate (PEGDMA, MW 750 g mol^−1^) and lithium phenyl-2,4,6-trimethylbenzoylphospinate (LAP, C16H16LiO3P) were purchased from Sigma-Aldrich (St. Louis, MO, USA) and used as received. Dimethylsulfoxide (DMSO) and ethanol (EtOH), purchased from Sigma-Aldrich (St. Louis, MO, USA), were used as solvent for LAP and plasticizer agent during gelation process, respectively.

### 4.2. Synthesis of tBA/PEGDMA/PEGMEMA Samples

Hydrogels with different tBA/PEGDMA/PEGMEMA weight ratios were obtained, carrying out all the preparation steps under nitrogen. Disc samples (40 mm diameter and 7 mm thickness) were prepared using cylindrical molds made in PTFE, to allow easier removal of the samples after polymerization and high chemical resistance.

Weighted amounts of PEGDMA and PEGMEMA were mixed in a vial and heated at 50 °C to allow PEGMEMA melting. In a second vial, the required amount of LAP was dissolved in DMSO leading to a transparent and slightly yellowish solution to which tBA was added. The solution was mixed with the PEGDMA-PEGMEMA mixture, and an appropriate amount of ethanol was added before casting into the molds. The specimens, placed in a PMMA box under flowing nitrogen, were photopolymerized using a Zeiss HBO 50/ac lamp (intensity 121 W/m^2^, measured by a Quantum Photoradiometer DO9721 DeltaOhm). The lamp was placed 15 cm from the samples. After gelation (typically occurring in less than 60 s), samples were removed and swollen in water for 24 h to wash out DMSO, changing water after the first 12 h. The specimens were then dried in an oven at 70 °C for 24 h.

The composition of the obtained samples is reported in Table 1. The samples are labeled according to tBA, PEGDMA and PEGMEMA weight percentages, for instance, sample 721 is composed of 70%wt of tBA, 20%wt of PEGDMA and 10%wt of PEGMEMA.

### 4.3. Characterization

For evaluating the water uptake/swelling behavior of the hydrogels, the specimens were soaked in water at 37 °C and weighted after 3 h and 7 days and then dried in oven at 70 °C for 24 h and weighted again. Measures were taken in triplicate. The swelling ratio (*sw*) and water content (*wc*) were, respectively, calculated according to the following equations:

(1) $$sw=\frac{\left(W_{SW}-W_{d}\right)}{W_{d}}$$

(2) $$wc=\frac{\left(W_{SW}-W_{d}\right)}{W_{SW}}$$

where *W_sw_* and *W_d_* are the weight of the swollen and the dry hydrogel, respectively.

Differential scanning calorimetry (DSC) analyses were performed on Q20 TA Instruments in nitrogen atmosphere with ramp temperature set to 10 °C/min, performing a double scan ranging from −10 to 100 °C, with the first one aiming at resetting the thermal history of the sample and eliminate volatile residuals of reaction.

Dynamic shear measurements were recorded to assess the rheological behavior of studied compositions. The samples were analyzed through a plate-to-plate rheometer (DHR2, TA Instruments). The linear range was detected through a strain sweep test performed in a range from 0.01 to 10% at 10 Hz. The rheological tests were performed on samples previously left soaking in water for 3 h. Both soaking and measurements were performed at 37 °C. Frequency sweep analyses were carried out by applying 0.1% strain in the 0.1 to 100 Hz range, after applying a preload of 35 N.

Solid-state nuclear magnetic resonance (SS-NMR) analyses were carried out with a Bruker Avance 400 WB spectrometer. NMR spectra were acquired with cross-polarization (CP) pulse sequence under the following conditions: ^13^C frequency, 100.48 MHz; π/2 pulse, 4.4 µs; decoupling length, 5.9 µs; recycle delay, 3 s; number of scans, 2 k; and contact time, 1.5 ms. The samples were packed in 4 mm zirconia rotor and spun at 8 kHz under air flow. Adamantane was used as external secondary reference. Pure liquid reagents were spun at 1 kHz and the spectra were recorded under low-power decoupling single-pulse sequence.

Moreover, molecular dynamics NMR measurements were performed through the collection of variable contact time experiments for each sample (1–4 ms, 12 points and spin-lock field strength equivalent to 63 kHz). The CP spectrum intensity depends on two competing factors, namely, the magnetization transfer from proton to carbon by dipolar coupling and the relaxation due to interaction with the local environment; the magnetization trend can be usually fitted with Equation (3):

M(t) = M_0_·e^−t/T1ρ(H)^·(1 − e^−t/TCH^) (3)

where M(t) is the peak intensity as a function of contact time t, M_0_ is the normalization constant, T_1ρ(H)_ is the proton spin–lattice relaxation time in the rotating frame and T_CH_ is the cross-polarization time constant. Thus, measuring spectrum intensity (M) as a function of CP contact time (t), it is possible to extract two parameters: the cross-polarization rate constant T_CH_ and the spin–lattice relaxation time T_1ρ(H_). Numerical estimation of T_CH_ and T_1ρ(H)_ values are usually obtained by fitting the experimental curve with single or multiple exponential laws according to homogeneity, segregation and/or domain size [43,44], thus describing the molecular motions of specific functional groups on typical time scales.

## Figures and Tables

**Table 1:** List of samples and relative tBA, PEGDMA and PEGMEMA percentage composition.

Sample Label	Nominal Composition, wt %		
	*tBA*	*PEGDMA*	*PEGMEMA*
811	80	10	10
721	70	20	10
631	60	30	10
541	50	40	10
712	70	10	20
622	60	20	20
532	50	30	20
442	40	40	20

## Data Availability

All data and materials are available on request from the corresponding author. The data are not publicly available due to ongoing research using a part of the data.

## Supplementary Materials

The following supporting information can be downloaded at https://www.mdpi.com/article/10.3390/gels9110912/s1, Figure S1: As-prepared samples; Figure S2: DSC diagrams of 721, 712, 631, 541 and 532. Arrows and color code are the ones described in the caption of Figure S2a. For better clarity, light orange solid arrows indicate first heating, blue are used for cooling and dark orange dashed ones for second heating scan; Figure S3: Superposition of ^13^C CPMAS spectra of sample 721 both fresh and after 5 months of storage at uncontrolled T and RH% of the laboratory. It is representative of all the samples; Figure S4: Profile fitting of the 55–20 ppm region for samples 622 and 811 after polymerization; peak V of the tBA at 28 ppm has been also used for optimizing the fitting. The assignments are presented in the table on the right; Figure S5: ^13^C variable contact time NMR magnetization curves of the resonances at 173 and 40 ppm for all the samples; Table S1: DSC main data for the tBA, PEGDMA and PEGMEMA. T_m_, T_m2_ melting temperature, L_f_ and L_f,2_ latent heat of fusion (first and second run, respectively), T_ev_ starting and T_ev_ peak starting and peak of evaporation and h_ev_ relative specific evaporation heat; Table S2: Sample labels, linewidth of the C=O resonance and branching degree (Equations (S1) and (S2)).
