# Supplementary material for: Polymeric Hydrogels for Intervertebral Disc Replacement/Integration: Playing with the Chemical Composition for Tuning Shear Behavior and Hydrophilicity
Source: Gels. 2023 Nov 17;9(11):912. doi: 10.3390/gels9110912 (PMC10671477; doi:10.3390/gels9110912)
Supplement: Supplementary file 1 [file gels-09-00912-s001.zip › gels-2700455-supplementary.pdf]

gels

Supplementary Materials

Polymeric Hydrogels for Intervertebral Disc Replacement/Integration: Playing with the Chemical Composition for Tuning Shear Behavior and Hydrophilicity

Devid Maniglio [1], Elia Bissoli [1,2], Emanuela Callone [2,*], Sandra Dirè [2,*] and Antonella Motta [1]

[1] BIOtech Research Center, Department of Industrial Engineering, University of Trento, Via Delle Regole 101, 38123 Trento, Italy; devid.maniglio@unitn.it (D.M.); bissolielia@gmail.com (E.B.); antonella.motta@unitn.it (A.M.)
[2] "Klaus Müller" Magnetic Resonance Lab, Department of Industrial Engineering, University of Trento, Via Sommarive 9, 38123 Trento, Italy
* Correspondence: emanuela.callone@unitn.it (E.C.); sandra.dire@unitn.it (S.D.)

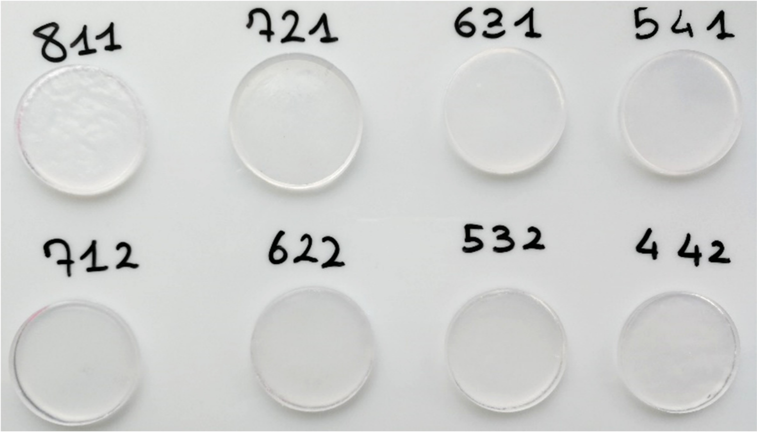

Figure S1. As prepared samples.

Table S1. DSC main data for the tBA, PEGDMA and PEGMEMA. T_m, T_{m2} melting temperature, L_f and $L_{f,2}$ latent heat of fusion (first and second run, respectively), $T_{ev\ starting}$ and $T_{ev\ peak}$ starting and peak of evaporation, and h_{ev} relative specific evaporation heat.

	tBA	PEGDMA	PEGMEMA
T_m (°C)		3.5	35.8
L_f (W/g)		52.5	131.3
$T_{ev\ starting}$ (°C)	24.1	33.9	
$T_{ev\ peak}$ (°C)	91.7	70.1	
h_{ev} (W/g)	106.8	18.8	
$T_{m,2}$ (°C)		6.6	35.8
$L_{f,2}$ (W/g)		10.3	131.3

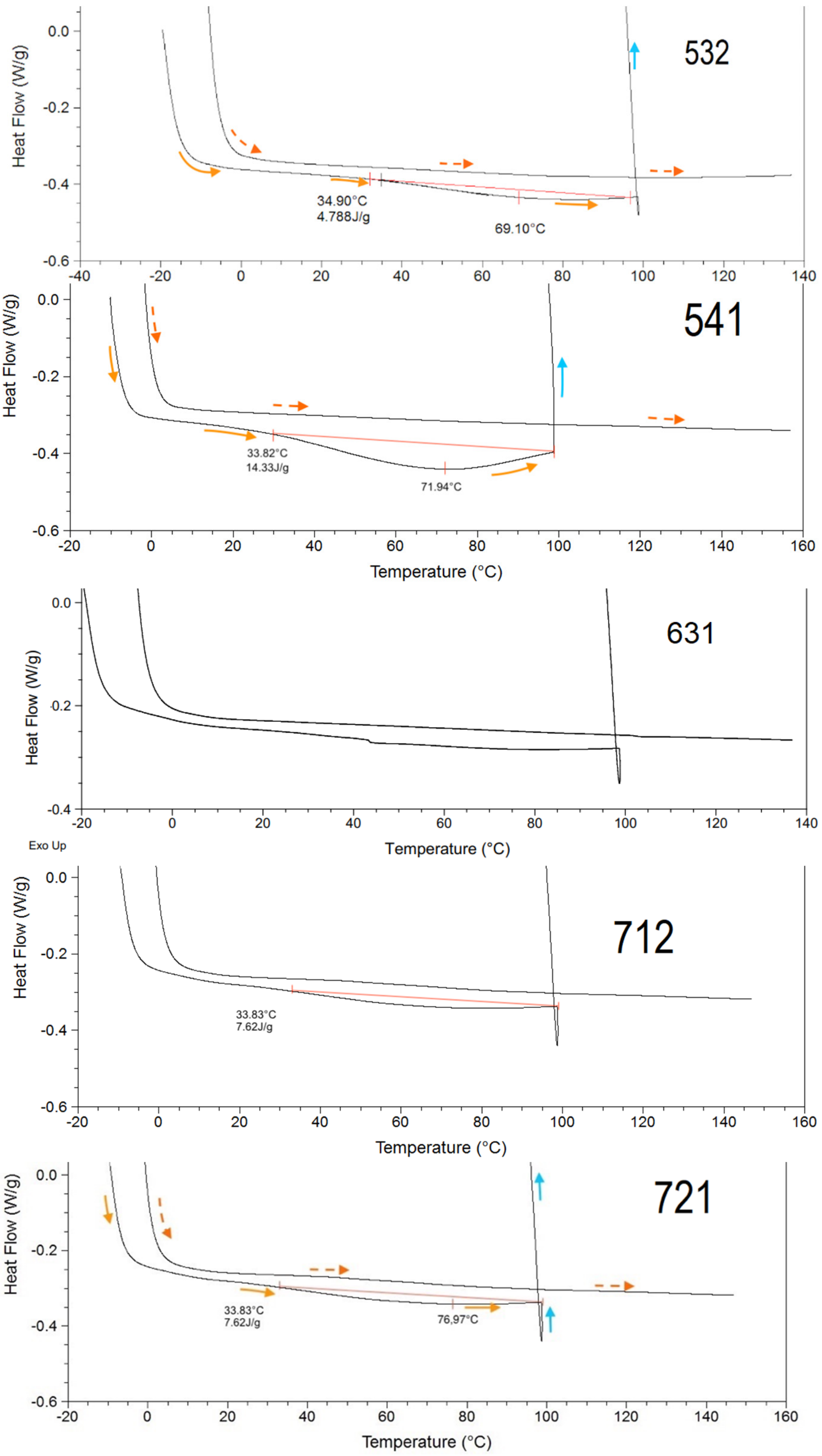

Figure S2. DSC diagrams of 721, 712, 631, 541and 532. For better clarity light orange solid arrows indicate first heating, blue are used for cooling and dark orange dashed ones for second heating scan.

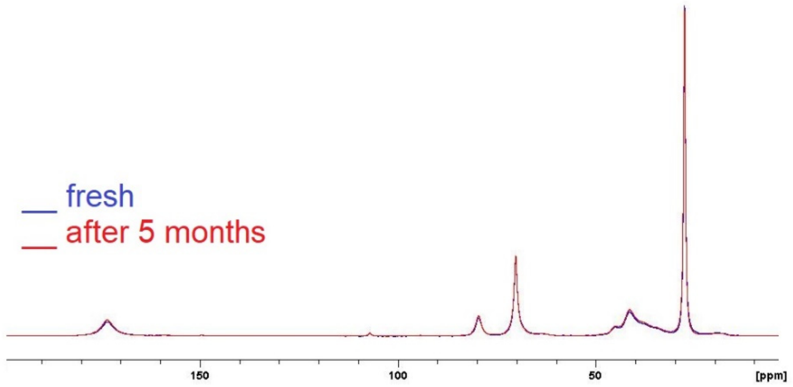

Figure S3. Superposition of ^{13}C CPMAS spectra of sample 721 both fresh and after 5 months of storage at uncontrolled T and RH% of the laboratory. It is representative of all the samples.

Details on the molecular structure organization of photo-polymerized hydrogels

As stated in par. 3.4 the tBA polymerization produces a complex resonance in the 50-30 ppm region, since all the functional groups created after the polymerization give rise to a specific resonance. This region could be separated into five resonances (as reported in Figure 4) and, for the sake of understanding, Scheme 1 is provided in the main text for the purpose of identifying the signals discussed, but should not be considered a representation of the actual structure of the hydrogel, which cannot be drawn with the acquired results.

In Figure S4 the five peaks are identified by capital letters: the components at 48, 41, 39 and 35 ppm are respectively assigned to quaternary carbons (**A**), main chain methine (**C**), methylenes adjacent to branch (**D**), and main chain CH_2 sites (**E**), respectively. Sites **D** and **E** take into account also the formed CH_2 groups of PEGMEMA and PEGDMA, and their quaternary carbon (**2'/b'**) is also detectable at 45 ppm (site **B)**.

Figure S4 shows two examples. The assignment has been made by comparison of all the spectra, and according to the literature [48,50]. For an accurate definition of these chemical shifts, the values were further compared with high resolution ^{13}C NMR and related DEPT spectra acquired on samples mildly swollen in THF (2-3 drops added to the rotor filled with the hydrogel).

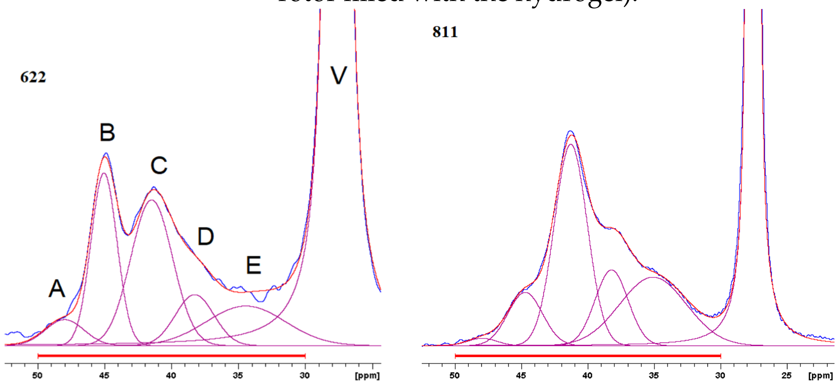

peak	assignment
A	Branch C_q (II')
B	Main chain C_q (2',b')
C	Main chain CH (II')
D	CH_2 close to branch(III',3',c')
E	Main chain CH_2 (III',3',c')

Figure S4. Profile fitting of the 55-20 ppm region for the sample 622 and 811 after polymerization; peak **V** of the tBA at 28 ppm has been also used for optimizing the fitting. The assignments are presented in the table on the right.

The degree of branching with the related error have been calculated as indicated in Castrignolles and Barrenetxe [48,50]. This approach takes into account that the maximum degree of branching could be theoretically obtained if all the CH of tBA (i.e peak **II'**) are

converted into quaternary carbons, since the polymerization results complete in all the cases. Equation S1 results the most reliable among those proposed in Castrignolles [48], although all of them give similar results in the present case.

$$\mathrm{DB\%} = A * 100/(A + C + D + E) \quad \text{(S1)}$$

$$\mathrm{SD\%} = \frac{100}{SNR} * \sqrt{[Df_{CDE}{}^{2} * A^{2} + Df_{A}{}^{2} * (C + D + E)^{2}]}/[Df_{A}{}^{2} * (2A + C + D + E)^{2}]$$

(S2)

The DB% is calculated taking into account the area of A, C, D, E peaks (Figure S4). Relative standard deviations, calculated by Equation S2, consider the parameters Df_{A} and Df_{CDE} that are the widths of the spectral integration range for A, C, D, E signals. The signal-to-noise ratio was calculated using the SNR calculation script was calculated with Topspin3.2 routine on a 10 ppm wide noise region. The calculated values are reported in Table S2 and shown in Figure S4.

Table S2. Sample labels, linewidth of the C=O resonance and branching degree (eq. S1 and S2).

1	2	3
**Sample label**	**C=O LW, Hz**	**DB% ± 1.0**
**811**	304	3.3
**721**	380	4.8
**631**	584	17.3
**541**	657	16.1
**712**	483	11.5
**622**	660	19.3
**532**	537	14.7
**442**	418	4.5

(a) (b)

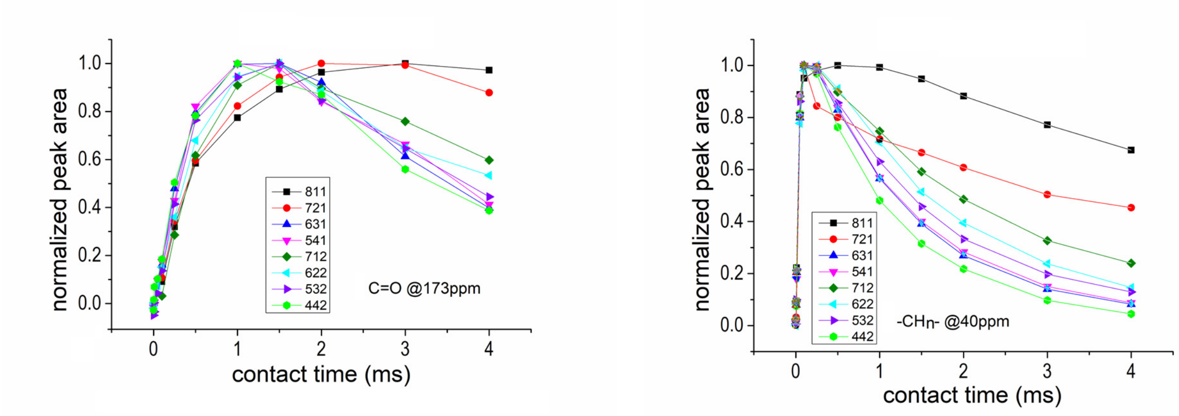

Figure S5. ^{13}C variable contact time NMR Magnetization curves of the resonances at 173 (a) and 40 ppm (b) for all the samples.

References

48. Castrignolles, P.; Graf, R.; Parkinson, M.; Wilhelm, M.; Gaborieau, M. Detection and quantification of branching in polyacrylates by size-exclusion chromatography (SEC) and melt-state 13C NMR spectroscopy. *Polymer* **2009**, *50*, 2373–2383. https://doi.org/10.1016/j.polymer.2009.03.021.

50. Barrenetxe, M.; Agirre, A.; Santos, J.I.; Badía, A.; Leiza, J.R.; Barquero, A. Oil-Based versus Bio-Based C8 Alkyl Chain (Meth)Acrylates in Emulsion Polymerization: Kinetics and Microstructure. *Macromol. React. Eng.* **2022**, *16*, 2200014. https://doi.org/10.1002/mren.202200014
